# Transfusion of standard-issue packed red blood cells induces pulmonary vasoconstriction in critically ill patients after cardiac surgery—A randomized, double-blinded, clinical trial

**DOI:** 10.1371/journal.pone.0213000

**Published:** 2019-03-11

**Authors:** Joanna Baron-Stefaniak, Gerda C. Leitner, Nina K. I. Küntzel, Elias L. Meyer, Michael J. Hiesmayr, Roman Ullrich, David M. Baron

**Affiliations:** 1 Department of Anaesthesia, Intensive Care Medicine and Pain Medicine, Medical University of Vienna, Vienna, Austria; 2 Department of Blood Group Serology and Transfusion Medicine, Medical University of Vienna, Vienna, Austria; 3 Section for Medical Statistics, Center for Medical Statistics, Informatics, and Intelligent Systems, Medical University of Vienna, Vienna, Austria; Public Library of Science, UNITED KINGDOM

## Abstract

**Background:**

Experimental and volunteer studies have reported pulmonary vasoconstriction during transfusion of packed red blood cells (PRBCs) stored for prolonged periods. The primary aim of this study was to evaluate whether transfusion of PRBCs stored over 21 days (standard-issue, siPRBCs) increases pulmonary artery pressure (PAP) to a greater extent than transfusion of PRBCs stored for less then 14 days (fresh, fPRBCs) in critically ill patients following cardiac surgery. The key secondary aim was to assess whether the pulmonary vascular resistance index (PVRI) increases after transfusion of siPRBCs to a greater extent than after transfusion of fPRBCs.

**Methods:**

The study was performed as a single-center, double-blinded, parallel-group, randomized clinical trial. Leukoreduced PRBCs were transfused while continuously measuring hemodynamic parameters. Systemic concentrations of syndecan-1 were measured to assess glycocalyx injury. After randomizing 19 patients between January 2014 and June 2016, the study was stopped due to protracted patient recruitment.

**Results:**

Of 19 randomized patients, 11 patients were transfused and included in statistical analyses. Eight patients were excluded prior to transfusion, 6 patients received fPRBCs (10±3 storage days), whereas 5 patients received siPRBCs (33±4 storage days). The increase in PAP (7±3 vs. 2±2 mmHg, P = 0.012) was greater during transfusion of siPRBCs than during transfusion of fPRBCs. In addition, the change in PVRI (150±89 vs. -4±37 dyn·s·cm^-5^·m^2^, P = 0.018) was greater after transfusion of siPRBCs than after transfusion of fPRBCs. The increase in PAP correlated with the change of systemic syndecan-1 concentrations at the end of transfusion (R = 0.64,P = 0.034).

**Conclusion:**

Although this study is underpowered and results require verification in larger clinical trials, our findings suggest that transfusion of siPRBCs increases PAP and PVRI to a greater extent than transfusion of fPRBCs in critically ill patients following cardiac surgery. Glycocalyx injury might contribute to pulmonary vasoconstriction associated with transfusion of stored blood.

## Introduction

Current transfusion practice allows for storage of packed red blood cells (PRBCs) up to 42 days, with the oldest compatible PRBC unit being assigned for transfusion (standard-issue PRBCs, siPRBCs). Observational studies indicate that transfusion of PRBCs stored for prolonged periods increases the risk of kidney injury and infection, and prolongs the length of hospital stay and time to extubation in patients undergoing cardiac surgery [1–3]. Furthermore, a meta-analysis in this patient population revealed that transfusion of stored PRBCs increases mortality risk [4]. In contrast, recent randomized controlled trials suggest that transfusion of siPRBCs does not negatively affect survival in a general population of hospitalized patients [5], in critically ill patients [6], or in patients following cardiac surgery [7].

Experimental and volunteer studies, however, reported a pulmonary vasoconstrictor effect of PRBCs stored for prolonged periods [8–12]. Baron et al. demonstrated that transfusion of PRBCs stored for 40 days transiently increases pulmonary artery pressure (PAP) and pulmonary vascular resistance (PVR) in lambs [8,9]. This pulmonary vasoconstrictor effect of stored PRBCs was potentiated by endothelial dysfunction induced by inhibition of nitric oxide (NO) synthesis [8] or hemorrhagic shock [9]. Furthermore, Berra et al. showed that transfusion of autologous PRBCs stored for 40 days increases PAP in obese adults with endothelial dysfunction [11]. We hypothesized that transfusion of stored PBRCs but not fresh PRBC would induce pulmonary vasoconstriction in critically patients following cardiac surgery.

Reduced NO synthesis by endothelial cells is a key characteristic of endothelial dysfunction, which is commonly diagnosed in patients undergoing cardiac surgery [13,14]. Several studies have associated the reduction of NO bioavailability with scavenging of NO by free hemoglobin (Hb) [15–20]. This effect was suggested as a central mechanism for vasoconstriction after transfusion of stored PRBCs [21,22]. However, additional factors might contribute to reduced bioavailability of NO. Blood transfusion induces degradation of the endothelial glycocalyx layer [23], which functions as a mechanical sensor and transducer used to regulate NO production by endothelial cells [24]. An injury of the glycocalyx during transfusion might further reduce NO bioavailability and potentiate the vasoconstrictor effects of PRBCs in patients with endothelial dysfunction. Furthermore, macrophage migration inhibitory factor (MIF), a mediator of the innate inflammatory response, has been associated with pulmonary vasoconstriction [25]. As transfusion of stored PRBCs induces innate inflammatory response, transfusion-related secretion of MIF might contribute to the pulmonary vasoconstrictor effects of stored PRBCs [26].

In this study we evaluated whether transfusion of PRBCs stored for more than 21 days (standard issue PRBCs, siPRBCs) would increase PAP and subsequently PVR to a greater extent than transfusion of PRBCs stored for less then 14 days (fresh PRBCs; fPRBCs) in critically ill patients following cardiac surgery. In addition, we assessed glycocalyx injury and release of MIF into serum after transfusion of fPRBCs and siPRBCs.

## Methods

This study was performed as a double-blinded, parallel-group, randomized clinical trial at the General Hospital of Vienna in accordance with the ethical standards stated in the Declaration of Helsinki. The CONSORT (Consolidated Standards of Reporting Trials) guidelines were used to guide the preparation of this article. Ethical approval for this study (reference number 1239/2013) was provided by the Ethical Committee of the Medical University of Vienna, Vienna, Austria. The study was registered at clinicaltrials.gov (NCT02050230) on January 17, 2014. Written informed consent was obtained from each patient before enrollment. Patients were screened for enrollment from January 2014 until June 2016.

### Study population

Patients (≥18 years) admitted to an intensive care unit (ICU) after cardiac surgery with a pulmonary arterial catheter in place and requiring transfusion of one PRBC unit as standard care were eligible for inclusion. Transfusion trigger was a Hb below 8 mg/dl. Exclusion criteria were ongoing hemodynamic support with adrenaline or continuous infusion of more than 0.2 μg·kg^-1^·min^-1^ noradrenaline, immediate need of transfusing 2 or more PRBC units, and administration of inhaled NO, prostacycline, or systemic phosphodiesterase inhibitors within 24 hours prior to inclusion.

According to the definition of a full analysis set defined by ICH E9 we excluded 8 patients, who were randomized without receiving treatment, in our case transfusion. Since no data were obtained and recorded for these patients, they were handled the same way as individuals not enrolled in the study.

### Study design, randomization and blinding

Patients were randomly assigned to receive transfusion of 1 unit of PRBCs stored <14 days (fPRBCs) or 1 unit of PRBCs stored for 21–42 days (siPRBCs). Packed red blood cells were leukoreduced and stored in saline-adenine-glucose-mannitol solution. Standard-issue PRBCs were handled as ‘first in, first out’, meaning that the oldest compatible unit from the blood bank was assigned for transfusion.

Allocation of PRBCs was performed at the Department of Blood Group Serology and Transfusion Medicine. The allocation list was generated using a computer-based random number generator (Randomizer, Social Psychology Network, Middletown, CT). Sequentially numbered envelopes with concealed allocation to respective study groups were prepared using a randomization ratio of 1:1. After an order for a PRBC unit was made by a study investigator at the ICU, a member of the Department of Blood Group Serology and Transfusion Medicine opened the concealed enveloped and prepared an appropriate PRBC unit for the randomized patient according to the assigned study group. The product number and expiration date were concealed, and the PRBC unit was sent to the ICU via pneumatic tube transport. At the ICU, a study investigator collected the PRBC unit, performed a bed-side test, and initiated and supervised the transfusion. Duration of transfusion was standardized to 15 minutes in all patients. The study investigators, study participants, the ICU team, and the statisticians performing the analysis remained blinded to treatment allocation status until final statistical analysis.

### Hemodynamic measurements

The PAP, mean arterial pressure (Infinity Delta, Draeger, Luebeck, Germany), and cardiac output (Vigilance II monitor, Edwards Lifescience, Irvine, CA, USA) were monitored continuously. Indices of pulmonary vascular resistance (PVRI) and systemic vascular resistance (SVRI) and the cardiac index were calculated using standard formulas. Hemodynamic parameters were recorded prior to transfusion at baseline (BL), immediately following transfusion (time point T15), and at 60 minutes after initiation of transfusion (T60). To assess whether hemodynamic parameters at T15 and T60 differed from baseline, values recorded at T15 and T60 were subtracted from those at baseline (Δ15 and Δ60, respectively).

### Blood sampling and measurements

Arterial blood samples were collected at baseline, at T15, and at T60. In addition, samples were collected from the transfusion line of each PRBC unit immediately after transfusion. Blood samples were analyzed for standard laboratory parameters including leukocytes, platelets, bilirubin, lactate dehydrogenase, creatine kinase, creatinine, alkaline phosphatase, aspartate aminotransferase, and alanine aminotransferase at the central laboratory. Additionally, arterial blood samples were analyzed for blood Hb concentrations, pH, lactate, glucose, sodium, and potassium using an ABL800 Flex blood gas analyzer (Radiometer Medical, Copenhagen, Denmark). Furthermore, arterial and PRBC samples were centrifuged for 10 minutes at 1000 *g* and 4°C. Supernatants were stored at -80°C until analysis.

Glycocalyx injury was assessed by measuring concentrations of syndecan-1 (SDC), a marker of glycocalyx degradation, in serum and in supernatants of PRBC units [27]. In addition, concentrations of the pro-inflammatory cytokine MIF were measured in serum and in supernatants of PRBC units. Concentrations of SDC and MIF were measured with enzyme-linked immunosorbent assays (ELISA) according to the manufacturer’s protocol (R&D Systems, Minneapolis, MN, USA). Furthermore, blood samples and supernatants of PRBC units were analyzed for concentrations of free Hb at the central laboratory. The changes in systemic concentrations of SDC, MIF, and free Hb between baseline and T15 (Δ15) were calculated by subtracting the concentrations at T15 from those at baseline.

### Sample size and study termination

Based on previous studies [8,11] we estimated that PAP would increase by 1±4 mmHg during transfusion of fPRBCs and by 4±4 mmHg during transfusion of siPRBCs. Using a two-group t-test with a 0.05 two-sided significance level, a sample size of 29 in each group was calculated to have 80% power to detect a difference in means of 3 mmHg assuming a common standard deviation of 4 mmHg. Taking into account an expected drop-out rate of 10%, we planned to include 64 patients in the study.

Despite a precise calculation of sample size based on previous data, we experienced unexpected protraction in patient recruitment after study initiation. Reasons for protracted patient recruitment were a reduced used of pulmonary arterial catheters in cardiac surgery implemented in 2014 at our institution, and a quick removal of pulmonary arterial catheters at the ICU when patients were stable following surgery. Furthermore, the concept of patient blood management was implemented at our department starting in 2014. A restrictive postoperative transfusion policy and the use of autologous transfusions devices during surgery resulted in a significant reduction of PRBCs transfused. These unforeseen developments markedly delayed realization and finalization of the study, which led the investigators to terminate the study in June 2016.

### Statistical analysis

The primary endpoint was the increase in PAP during transfusion of PRBCs (Δ15 PAP) with the increase in PVRI during transfusion of PRBCs (Δ15 PVRI) as the key secondary end point. Welch’s t-tests was performed to analyze whether Δ15 PAP and Δ15 PVRI during transfusion of fPRBCs differed from those during transfusion of siPRBCs. Additionally, an ANCOVA using the baseline value as a covariate was computed and results were compared to those of the Welch’s t-tests.

Secondary endpoints: Differences in all other measured hemodynamic parameters among groups were assessed using Welch’s t-tests. Concentrations of SDC, MIF and free Hb in serum and in the supernatant of PRBCs were analyzed with Welch’s t-tests to compare differences between groups. Correlations between Δ15 PAP and Δ15 of SDC, MIF and free Hb in serum and supernatants of PBRCs were calculated using Pearson’s correlation coefficient. Asymptotic 95% confidence intervals for correlation coefficients were calculated using Fisher’s z-transformation.

Data at any study level were obtained and recorded only for randomized and transfused patients, but not for randomized patients who were not transfused. Hence, these individuals were not included in analyses. A value of P<0.05 was considered statistically significant. All P-values cited in the manuscript are derived from Welch’s t-tests, while P-values derived from ANCOVAS are reported in the supporting material only. Calculated P-values for secondary endpoints serve only descriptive purposes, hence no multiple testing corrections were applied. Statistical analysis was performed using SPSS 23 (SPSS Inc., Chicago, IL, USA) and R 3.3.2.

## Results

**Fig 1:**
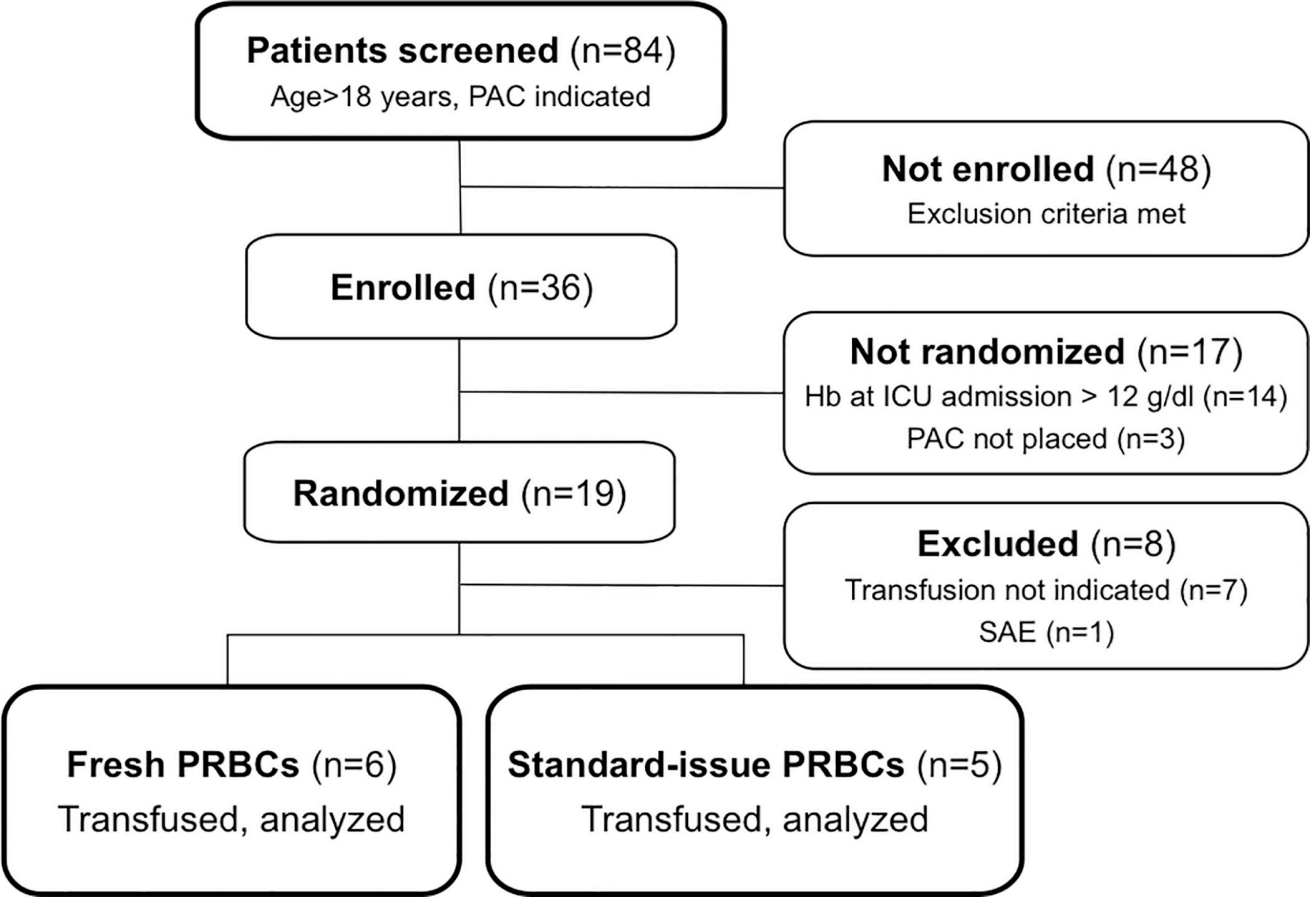
Study participation flow diagram. Abbreviations: PAC, pulmonary artery catheter; Hb, hemoglobin; ICU, intensive care unit; SAE, serious adverse event; PRBCs, packed red blood cells.

Nineteen patients were randomized between January 2014 and June 2016, of which 7 patients were not transfused and excluded prior to study procedures and measurements (Fig 1). One patient developed a severe adverse event due to a hemolytic reaction (from which she fully recovered) and was excluded from analysis. Eleven patients completed the study and were included in the final analysis.

Of the patients included in the analysis, 6 patients received fPRBCs, whereas 5 patients received siPRBCs. Mean storage duration was 10±3 days for fPRBCs and 33±4 days for siPRBCs. Characteristics and demographic data of study participants and performed surgical procedures are depicted in Table 1. Baseline laboratory parameters of both groups are listed in S1 Table.

**Table 1:** Demographic and baseline characteristics of the study population. Data are depicted as absolute numbers or mean±standard deviation. Abbreviations: APACHE, acute physiology and chronic health evaluation; CABG, coronary artery bypass grafting; COPD, chronic obstructive pulmonary disease; PRBCs, packed red blood cells.

Parameter	Fresh PRBCs	Standard-issue PRBCs
*Demographic data*		
Patients (n)	6	5
Female/male (n)	1/5	2/3
Ethnicity (n, Caucasian/Other)	6/0	5/0
Age (years)	71±7	69±6
Body-mass index (kg/m^2^)	29±7	25±3
APACHE II Score	17±5	19±6
*Comorbidities (n)*		
Arterial hypertension	5	5
Coronary artery disease	3	3
Diabetes	2	2
Cardiomyopathy	2	2
Pulmonary artery hypertension	1	0
COPD	1	1
*Surgical characteristics*		
Procedure (n)		
CABG	1	1
CABG plus aortic valve replacement	1	1
Aortic valve replacement	2	2
Mitral valve replacement	1	1
CABG plus mitral valve replacement	1	0
Total time in operating theatre (hours)	9±2	8±2
*Time between surgery and study transfusion (hours)*	70	74
*Data prior to study transfusion*		
Hemoglobin (mg/dl)	8.0±0.7	8.2±0.6
Positive end-expiratory pressure ventilation (n)	2	1
Vasopressor use (n)	6	5
Noradrenaline	2	3
Noradrenaline plus Dobutamine	4	2

### Primary and key secondary endpoint

**Fig 2:**
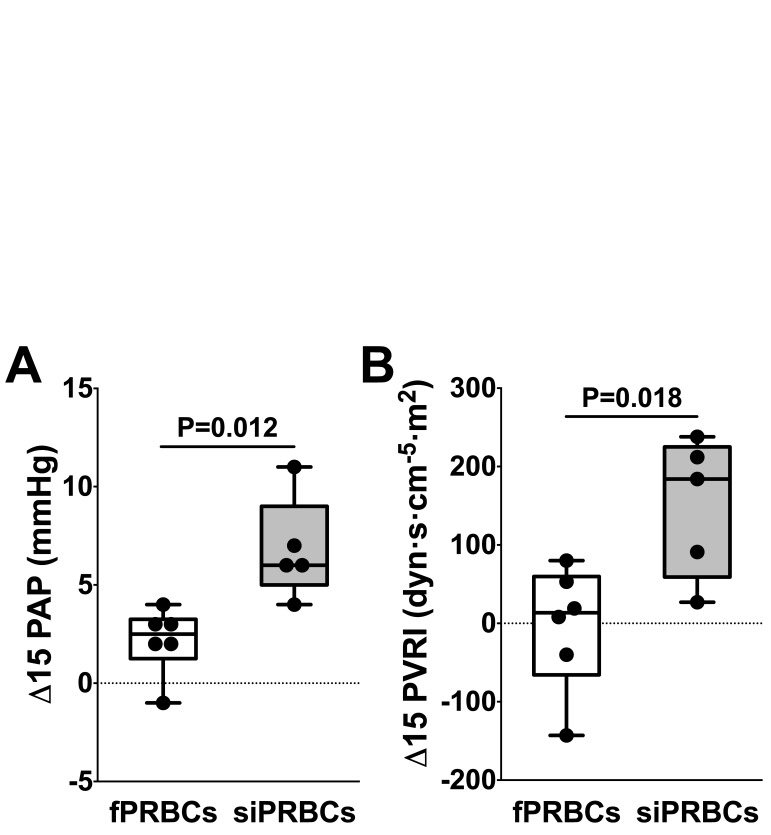
Changes in pulmonary arterial pressure and pulmonary vascular resistance index at 15 minutes after initiation of transfusion. Change in (A) pulmonary arterial pressure (Δ PAP) and (B) pulmonary vascular resistance index (Δ PRVI) at 15 minutes after initiation of transfusion in patients receiving fresh packed red blood cells (fresh PRBCs, white bars) or standard-issue packed red blood cells (standard-issue PRBCs, grey bars). Black dots represent respective changes in individual patients. P-values indicate differences among patients receiving fresh PRBCs and standard-issue PRBCs.

The increase in PAP (Δ15 PAP) was greater during transfusion of siPRBCs than during transfusion of fPRBCs (P = 0.012, 95% CI for difference in means [-7.86; -1.41]; Fig 2A and Table 2). In addition, the increase in PVRI (Δ15 PVRI) was greater during transfusion of siPRBCs than during transfusion of fPRBCs (P = 0.016, 95% CI for difference in means [-272; -37]; Fig 2B and Table 2). Correspondingly, results of ANCOVA demonstrate a greater increase in PAP and PVRI after transfusion of siPRBCs than after transfusion of fPRBCs (S2 Table).

**Table 2:** Hemodynamic parameters at baseline, and within 15 minutes and 60 minutes after transfusion. P-values (Welch’s t-Test) indicate differences versus baseline within each group. Abbreviations: BL, baseline; CI, cardiac index; MAP, mean arterial pressure; PAP, pulmonary arterial pressure; PRBC, packed red blood cells; PVRI, pulmonary vascular resistance index; SVRI, systemic vascular resistance index.

	Fresh PRBCs		Standard-issue PRBCs	
Time point	Parameter	P-value	Parameter	P-value
	PAP (mmHg)		PAP (mmHg)	
BL	29±5		24±3	
15	31±6	0.651	31±3	**0.039**
60	29±4	0.822	27±3	0.321
	PVRI (dyn·s·cm^-5^·m^2^)		PVRI (dyn·s·cm^-5^·m^2^)	
BL	394±144		257±101	
15	390±162	0.989	407±169	0.276
60	428±187	0.923	303±120	0.879
	MAP (mmHg)		MAP (mmHg)	
BL	80±10		76±10	
15	83±11	0.994	84±6	0.378
60	80±9	0.870	83±5	0.544
	SVRI (dyn·s·cm^-5^·m^2^)		SVRI (dyn·s·cm^-5^·m^2^)	
BL	2351±288		2349±1220	
15	2374±310	0.991	2652±1203	0.844
60	2505±759	0.947	2470±1070	0.972
	CI (l/min/m^2^)		CI (l/min/m^2^)	
BL	2.9±0.3		3.3±1.5	
15	2.9±0.3	0.967	3.1±1.2	0.988
60	2.8±0.5	0.942	3.3±1.3	0.944

### Secondary endpoints

The PAP increased from 24±3 mmHg at BL to 31±3 mmHg at T15 (P = 0.039) after transfusion of siPRBCs, but did not change after transfusion of fPRBC (29±5 mmHg at BL vs. 31±6 mmHg at T15, P = 0.651, Table 2). The PVRI values did not differ in patients receiving siPRBCs (257±101 dyn·s·cm^-5^·m^2^ at BL vs. 407±169 dyn·s·cm^-5^·m^2^ at T15 (P = 0.276), or those receiving fPRBCs (394±144 dyn·s·cm^-5^·m^2^ at BL vs. 390±162 dyn·s·cm^-5^·m^2^ at T15, P = 0.989, Table 2).

Changes in PAP and PVRI for Δ60 did not differ among groups (Table 3). The increase in SVRI (Δ15 SVRI) was greater during transfusion of siPRBCs than during transfusion of fPRBCs (P = 0.005). Furthermore, changes in cardiac index and mean arterial pressure for Δ15 and Δ60, and SVRI for Δ60 did not differ among groups. Changes in hemodynamic parameters between baseline and T15 are depicted in S2 Table.

**Table 3:** Changes of hemodynamic and laboratory parameters at 15 and 60 min following start of transfusion. Data are depicted as mean±standard deviation. P-values (Welch’s t-Test) indicate differences among patients receiving fresh and standard-issue PRBCs. Statistically significant data are printed bold. Abbreviations: Δ15, change in parameter during transfusion; Δ60, change in parameter within the first 60 minutes after starting transfusion; CI, cardiac index; Hb, hemoglobin; MIF, macrophage migration inhibitory factor; PAP, pulmonary arterial pressure; PRBCs, packed red blood cells; PVRI, pulmonary vascular resistance index; SDC, syndecan-1; SVRI, systemic vascular resistance index; MAP, mean arterial pressure; CI, cardiac index.

	Δ 15			Δ 60		
Parameter	Fresh PRBCs	Standard-issue PRBCs	P-value	Fresh PRBCs	Standard-issue PRBCs	P-value
PAP (mmHg)	**2±2**	**7±3**	**0.012**	1±2	3±1	0.079
PVRI (dyn·s·cm^-5^·m^2^)	**-4±79**	**150±89**	**0.016**	35±90	47±22	0.761
MAP (mmHg)	4±3	8±5	0.116	0±5	7±6	0.076
SVRI (dyn·s·cm^-5^·m^2^)	**25±140**	**303±106**	**0.005**	154±624	121±192	0.907
CI (L·min^-1^·m^2^)	0.1±0.2	-0.2±0.4	0.183	-0.1±0.3	0±0.3	0.540
SDC (ng/ml)	1±2	3±3	0.497	0±1	4±8	0.348
MIF (ng/ml)	-1±10	13±22	0.233	4±21	14±12	0.333
Free Hb (mg/dl)	1±1	8±8	0.141	1±1	4±5	0.185

Concentrations of SDC in the supernatant of fPRBCs and siPRBCs were below the detection limit of the ELISA kit. Concentrations of MIF in the supernatant of fPRBCs did not differ significantly from those in the supernatant of siPRBCs (158±115 vs. 521±436 ng/ml, respectively, P = 0.137). Similarly, concentrations of free Hb in the supernatant of fPRBCs units did not differ from those in the supernatant of siPRBCs (76±33 vs. 165±114 mg/dl, P = 0.158). No correlations were found between the increase in PAP (Δ15 PAP) and the concentrations of MIF (R = 0.26, 95% CI [-0.40; 0.74]) and free Hb (R = 0.22, 95% CI [-0.44; 0.72]) in the supernatant of PRBCs.

The increase in PAP (Δ15 PAP) positively correlated with the increase of systemic SDC concentrations (Δ15 SDC; R = 0.64, 95% CI [0.06; 0.90]). The increase in PAP during transfusion did not correlate with Δ15 MIF (R = 0.14, 95% CI [-0.50; 0.68]) or Δ15 free Hb (R = 0.17, 95% CI [-0.48; 0.70]).

## Discussion

In this double-blinded, randomized clinical study we investigated whether transfusion of PRBCs induces pulmonary vasoconstriction in critically ill patients following cardiac surgery. The study was terminated due to protracted patient recruitment and remained underpowered. In the 11 critically ill patients included in the study, transfusion of siPRBCs increased PAP and PVRI to a greater extent than transfusion of fPRBCs. Additionally, SVRI increased after transfusion of siPRBCs, but not after transfusion of fPRBCs. Mean arterial pressure and cardiac output did not differ among groups. Furthermore, the increase in PAP was associated with an increase in concentrations of SDC, a marker of glycocalyx injury.

In a volunteer study, Berra et al. confirmed that transfusion of stored PRBCs increases PAP in obese individuals with endothelial dysfunction. Because transfusion of stored PRBCs did not alter cardiac output, Berra et al. suggested that the increase in PAP was mediated by an increase in PVR caused by pulmonary vasoconstriction. The investigators reported an estimated PAP, which was calculated from the pulmonary artery acceleration time measured by transthoracic ultrasound [11]. In our study we measured the PAP directly using pulmonary artery catheters. Similarly to the findings reported by Berra et al. we found a greater increase in PAP and PVRI after transfusion of siPRBCs than after transfusion of fPRBCs. Additionally, the cardiac output remained unchanged in both groups. Although our results have to be interpreted with caution due to the small patient number included in this study, our findings support the assumption by Berra et al. that the increase in PAP after transfusion of stored PRBCs is caused by pulmonary vasoconstriction. However, the findings require verification in larger clinical trails.

In addition to pulmonary vasoconstriction, siPRBCs induced systemic vasoconstriction in our patients. These data are in agreement with findings by Risbano et al [28]. The authors transfused healthy volunteers with autologous leukopheresed PRBCs. Transfusion of PRBCs stored for 42 days decreased acetylcholine-dependent forearm blood flows to a greater extent than transfusion of PRBCs stored for 5 days. Similarly, Rezoagli and co-authors reported a transient increase in both pulmonary and systemic vascular resistances in patients developing hemolysis after cardio-pulmonary bypass [29]. These results indicate systemic vasoconstriction with significant impairment of endothelial function after transfusion of PRBCs stored for prolonged periods or prolonged periods of cardio-pulmonary bypass.

Endothelial dysfunction occurs in disorders including hypertension, diabetes, and atherosclerosis [30–32], and has been shown to augment vasoconstriction after transfusion of stored PRBCs in mice [33], in lambs [8], and in humans [11]. All patients included in our study suffered from disorders associated with endothelial dysfunction. Thus, it is likely that endothelial dysfunction was present in individuals included in our study. Reduced bioavailability of NO is one of the key characteristics of endothelial dysfunction. In healthy individuals, the glycocalyx functions as a mechanical sensor and transducer used to regulate NO production by endothelial cells [24]. Experimental studies have demonstrated that removal of glycocalyx components reduces cellular NO production upon mechanical stimuli [34,35]. In humans, Larsen et al. suggested that blood transfusion induces degradation of the endothelial glycocalyx layer after measuring elevated concentrations of SDC following blood transfusion [23]. Syndecan-1 is generally acknowledged as a marker of endothelial glycocalyx injury. In our study, the increase in PAP after transfusion of PRBCs positively correlated with systemic SDC concentrations. Based on this finding we speculate that glycocalyx injury might play a key role in the mechanism behind pulmonary vasoconstriction associated with transfusion of stored PRBCs.

Several studies associated the reduction of NO bioavailability with scavenging of NO by free Hb. Hemolysis increases during storage of PRBCs, and the host’s systemic concentrations of free Hb increase after transfusion of stored PRBCs [11,28,36]. Free Hb rapidly reacts with NO to form methemoglobin and nitrate, resulting in NO consumption [15,16]. In addition, hemolysis decreases NO bioavailability by releasing arginase-1, an enzyme converting arginine to ornithine [17,18]. Arginase-1 reduces concentrations of arginine, which is a substrate for NO synthesis. In our study, free Hb concentrations in serum did not differ among groups. The low number of patients included most likely explains the lack of statistical significance.

The pro-inflammatory cytokine MIF has previously been linked to pulmonary vasoconstriction. Clinical studies demonstrated elevated MIF concentration in patients suffering from pulmonary hypertension [25,37]. Animal studies showed that antibodies directed against MIF reversed hypoxia-induced pulmonary hypertension in mice and in rats [25,38]. Although Zhang et al. reported that MIF has no direct vasoconstrictor effects in the pulmonary circulation, the authors demonstrated that MIF potentiated pulmonary vasoconstriction pre-evoked by phenylephrine [38]. In our study, we were unable to confirm an association between MIF and pulmonary vasoconstriction after transfusion of stored blood. Concentrations of MIF in the supernatant of fPRBCs and siPRBCs did not differ significantly due to the limited patient numbers.

Current clinical practice is to transfuse the oldest compatible unit available at the blood bank. Some clinical studies investigating the effects of storage duration of PRBCs had only small differences in storage durations among groups. A strength of our study is the marked difference in storage duration of PRBCs (10±3 vs. 33±4 days). Furthermore, the inserted pulmonary arterial catheter allowed direct measurement of PAP and calculation of PVR.

There are several limitations to our study. A major limitation is the low number of included patients. During preparation of the study protocol in 2012, sample size calculation yielded that inclusion of 29 patients per group would allow to detect a significant difference in PAP with a power of 80%. However, after initiating the study in 2014, we experienced protracted patient recruitment starting from 2015. One reason for protracted patient inclusion was a reduced use of pulmonary arterial catheters in cardiac surgery enforced at our cardiac anesthesiology department since 2014. In addition, our hospital implemented the concept of patient blood management in 2014, which is a multidisciplinary approach focusing on reducing blood transfusions. In particular, anesthesiologists started to adhere to a restrictive transfusion policy in the postoperative phase and to use autologous cell salvage devices during surgery. These unforeseen developments made it impossible to achieve the planned patient number within a reasonable time frame. Therefore, we decided to stop the study in June 2016 and perform analysis with the included patients.

Of note, this low number of included and analyzed patients resulted in an underpowered study. With respect to scientific studies, low power means that the chance of discovering effects that are genuinely true is low [39]. Results from underpowered studies are at greater risk of being false negative than results from adequately powered studies. In addition, when underpowered studies discover true effects, the found effects might be inflated, resulting in a phenomenon called winner’s curse [39]. Thus, although statistic analysis yielded significant results in our study, the results should be interpreted as indefinite or unproven and require verification in larger studies.

Another limitation is that the absolute PAP values after transfusion did not differ between groups, and the increase in PAP was transient and not detectable within 60 minutes after transfusion. As a consequence, we were unable to answer whether the increase of PAP and PVR after transfusion of stored blood is clinically relevant and could translate into adverse pulmonary and cardiac events. Larger clinical trials are required to clarify whether an increase in PAP and PVR after transfusion of stored blood influences outcomes in patients following cardiac surgery. Furthermore, as only one unit of PRBCs was transfused, transfusion of multiple PRBC units might potentiate the increase in PAP and PVR and thus negatively affects outcome. In addition, we tried to identify new molecules potentially contributing to pulmonary vasoconstriction after transfusion of stored blood, but the limited number of samples analyzed does not allow us to draw significant conclusion from our results. In addition, we did not focus on pathomechanisms described in previous experimental settings. Thus, other possible noxious effects of stored PRBC transfusions mediated by iron, microparticles, protein oxidation, or lipid peroxidation of the erythrocyte membrane were not assessed in our study.

In conclusion, the results of this study suggest that transfusion of siPRBCs might increase PAP and PVRI to a greater extent than transfusion of fPRBCs in patients admitted to the ICU following cardiac surgery.

## Supporting information

S1 CONSORT checklist Checklist of information required for reporting a randomised trial. (DOC)

S1 Table Laboratory values of study participants prior to transfusion. Data are depicted as mean±standard deviation. Abbreviations: PRBCs, packed red blood cells.
(DOC)

S2 Table Results of ANCOVA for the increase within 15 minutes during transfusion as the dependent variable. Abbreviations: ANCOVA, analysis of co-variance; CI, cardiac index; FHB, free hemoglobin; MAP, mean arterial pressure; PAP, pulmonary arterial pressure; PRBC, packed red blood cells; PVRI, pulmonary vascular resistance index; SVRI, systemic vascular resistance index.
(DOC)

S1 Dataset Complete anonymized dataset of the study population. Abbreviations: AH, arterial hypertension; AS, aortic stenosis; AVR, aortic valve replacement; CABG, coronary artery bypass graft; CAD, coronary artery disease; CMP, cardiomyopathy; COPD, chronic obstructive pulmonary disease; FHB, free hemoglobin; GERD, gastro-esophageal reflux disease; HLP, hyperlipidemia; HR, heart rate; MAP, mean arterial pressure; MVR, mitral valve replacement; NIDDM, non insulin-dependent diabetes mellitus; PAP, pulmonary arterial pressure; PEEP, positive end-expiratory pressure; PVRI, pulmonary vascular resistance index; RBC, red blood cells; SVRI, systemic vascular resistance index.
(XLSX)

S1 Study Protocol English translation of the study protocol. (PDF)

S2 Study Protocol Original German version of the study protocol. (PDF)
