# Supplementary material for: Transfusion of standard-issue packed red blood cells induces pulmonary vasoconstriction in critically ill patients after cardiac surgery—A randomized, double-blinded, clinical trial
Source: PLoS One. 2019 Mar 11;14(3):e0213000. doi: 10.1371/journal.pone.0213000 (PMC6411146; doi:10.1371/journal.pone.0213000)
Supplement: S1 Table — Data are depicted as mean±standard deviation. Abbreviations: PRBCs, packed red blood cells. (DOC) [file pone.0213000.s002.doc]

**S1 Table. Laboratory values of study participants prior to transfusion**.

| **Parameter** | **Fresh**  **PRBCs** | **Standard-issue**  **PRBCs** |
| --- | --- | --- |
|  |  |  |
| Alanine aminotransferase (IU/L) | 13±4 | 30±27 |
| Aspartate aminotransferase (IU/L) | 42±32 | 97±108 |
| Lactate dehydrogenase (IU/L) | 291±118 | 440±200 |
| Bilirubin (mg/dL) | 2.2±2.0 | 1.3±1.0 |
| Lactate (mmol/L) | 1.3±0.3 | 1.6±0.6 |
| Creatine kinase (IU/L) | 301±190 | 445±261 |
| Creatinine (mg/dL) | 1.3±0.6 | 1.4±1.3 |
| Leukocytes (G/L) | 11±4 | 13±7 |
| Platelets (G/L) | 101±72 | 86±30 |
| Arterial pH | 7.40±0.05 | 7.40±0.03 |
| Glucose (mg/dL) | 143±42 | 161±23 |
| Potassium (mmol/L) | 4.1±0.6 | 4.7±0.4 |
| Sodium (mmol/L) | 139±2 | 139±2 |
|  |  |  |

Data are depicted as mean±standard deviation. Abbreviations: PRBCs, packed red blood cells.
