# Supplementary material for: Transfusion of standard-issue packed red blood cells induces pulmonary vasoconstriction in critically ill patients after cardiac surgery—A randomized, double-blinded, clinical trial
Source: PLoS One. 2019 Mar 11;14(3):e0213000. doi: 10.1371/journal.pone.0213000 (PMC6411146; doi:10.1371/journal.pone.0213000)
Supplement: S2 Table — Abbreviations: ANCOVA, analysis of co-variance; CI, cardiac index; FHB, free hemoglobin; MAP, mean arterial pressure; PAP, pulmonary arterial pressure; PRBC, packed red blood cells; PVRI, pulmonary vascular resistance index; SVRI, systemic vascular resistance index. (DOC) [file pone.0213000.s003.doc]

**S2 Table. Results of ANCOVA for the increase within 15 minutes during transfusion as the dependent variable.**

| **Variable** | **Intercept Coefficient** | **PRBC Storage Age** | |
| --- | --- | --- | --- |
|  |  | Coefficient | P Value |
| PAP | 5.04 | 4.15 | 0.009 |
| PVRI | -43.92 | 93.71 | 0.014 |
| FHB | 4.99 | 7.33 | 0.092 |
| MAP | 17.51 | 4.06 | 0.075 |
| SVRI | 25.51 | 158.89 | 0.006 |
| CI | 1.35 | -0.40 | 0.054 |
| Given P Values derive from ANCOVA analysis.  Abbreviations: ANCOVA, analysis of co-variance; CI, cardiac index; FHB, free haemoglobin; MAP, mean arterial pressure; PAP, pulmonary arterial pressure; PRBC, packed red blood cells; PVRI, pulmonary vascular resistance index; SVRI, systemic vascular resistance index. | | | |
