# Supplementary material for: Transfusion of standard-issue packed red blood cells induces pulmonary vasoconstriction in critically ill patients after cardiac surgery—A randomized, double-blinded, clinical trial
Source: PLoS One. 2019 Mar 11;14(3):e0213000. doi: 10.1371/journal.pone.0213000 (PMC6411146; doi:10.1371/journal.pone.0213000)
Supplement: S1 Study Protocol — (PDF) [file pone.0213000.s005.pdf]

MEDIZINISCHE UNIVERSITÄT WIEN

Comparison of hemodynamic changes after transfusion of fresh and stored red blood cells in critically ill patients after cardiac surgery

Dr. David Baron, Dr. Joanna Stefaniak and Ao.Univ.-Prof.Dr. Roman Ullrich

Department of Anesthesia, Critical Care and Pain Management, Medical University of Vienna, Waehringer Guertel 18-20, 1090 Vienna, Austria

Ass.Prof.Dr. Gerda Leitner

Department of Blood Group Serology and Transfusion Medicine, Medical University of Vienna, Waehringer Guertel 18-20, 1090 Vienna, Austria

Elias Meyer

Section for Medical Statistics, Center for Medical Statistics, Informatics, and Intelligent Systems, Medical University of Vienna, Austria

MEDIZINISCHE UNIVERSITÄT WIEN

Index

1 Background 3
2 Aims 3
3 Patients 4
4 Definition of study groups 4
5 Endpoints 5
5.1 Primary endpoint 5
5.2 Secondary endpoints 5
6 Methods 6
6.1 Study design 6
6.2 Randomization and blinding 6
6.3 Data and sample collection 6
7 Statistics 8
7.1 Sample size calculation 8
7.2 Hypothesis 8
7.3 Statistical analysis 8
8 Data protection 10
9 Ethical considerations and risk-benefit-evaluation 10
10 References 11

1 Background

Internationally, packed red blood cells (PRBCs) can be stored for up to 42 days. Prolonged storage of PRBCs induces various biochemical, pathophysiological and functional changes in red blood cells.[1,2]

Experimental study in lambs demonstrated that transfusion of PRBCs stored for 40 days induced pulmonary hypertension, while transfusion of fresh PRBCs stored for 2 days did not alter pulmonary hemodynamics.[3] The vasoconstrictive effects of stored PRBC were potentiated by preceding induction of hemorrhagic shock.[4] Also clinical studies suggest that patients with endothelial dysfunction are at risk of cardiopulmonary complications after transfusion of stored PRBC. Berra et al. showed that the pulmonary artery pressure (PAP) increases after transfusion of stored PRBC in obese adults with endothelial dysfunction, but not after transfusion of fresh PRBC.[5]

In this study we want to assess whether transfusions of stored PRBC, but not fresh PRBC, increases the PAP and PVR in critically ill patients following cardiac surgery. We further will study whether free hemoglobin concentrations in serum and PRBC units correlate with the increase of PAP after transfusion.

2 Aims

After observing induction of pulmonary vasoconstriction after transfusion of stored blood in lambs, we aim to assess whether transfusion of stored PRBCs induces pulmonary vasoconstriction in humans.

We further want to study whether free hemoglobin concentrations in serum and PRBC units correlate with the increase of PAP after transfusion. Additionally, we will evaluate whether transfusion of stored PRBC leads to greater glycocalx injury, and a greater release of MIF than transfusion of fresh PRBC.

3 Patients

Eligible patients will be critically ill adults after cardiac surgery (18 years or older) with a pulmonary arterial catheter in situ admitted to an intensive care unit (ICU) at the General Hospital of Vienna. An order for a PRBC transfusion requested by the study participant's health care team will be required before randomization.

Exclusion criteria:

- Age <18
- likely exitus within the next 24 hours
- acute bleeding requiring transfusion of more than 2 units PRBCs within the next hour
- continuous infusion of more than 0.2 µg/kg/min noradrenaline
- continuous infusion of adrenaline
- administration of inhalative NO or inhalative prostacyclin
- administration of phosphodiesterase-5-inhibitors within the last 24 hours prior enrollment
- sepsis

4 Definition of study groups

Packed red blood cells will be considered fresh when the unit was stored at the blood bank for 14 or less days, while stored PRBC were pooled at the blood bank for 21 to 42 days. Stored PRBC will be handled as 'first in, first out', meaning that the unit with the longest storage in the blood bank will be assigned to the compatible study patient randomized to receive stored PRBCs.

5 Endpoints

5.1 Primary endpoint

The primary endpoint was the increase in PAP during the first 15 minutes after transfusion of PRBCs (ΔPAP15).

5.2 Secondary endpoints

The secondary endpoints include:

- The increase in PAP during the first 60 minutes (ΔPAP60) and the increase in PVR, MAP, SVR, FHB and CO in the first 15 and 60 minutes (ΔPVR15, ΔPVR60, ΔMAP15, ΔMAP60, ΔSVR15, ΔSVR60, ΔFHB15, ΔFHB60, ΔCO15, ΔCO60).
- The correlation between ΔFHB15, ΔMIF15, FHB EK, MIF EK and ΔPAP15.
- MIF EK, SDC EK, MIF0, SDC0, MIF15, SDC15, MIF60, SDC60, FHB0.
- ΔFHB15, ΔFHB60, ΔMIF15, ΔMIF60, ΔSDC15, ΔSDC15.

6 Methods

6.1 Study design

This study will be performed as a double-blinded, parallel-group, randomized clinical trial at the Medical University of Vienna in accordance with the ethical standards stated in the Declaration of Helsinki.

6.2 Randomization and blinding

Patients will be randomly assigned for transfusion of 1 unit of stored PRBC or 1 unit of fresh PRBC at a 1:1 ratio. Allocation of PRBCs will performed at the Department of Blood Group Serology and Transfusion Medicine. After covering the product number and expiration date by the blood bank, the PRBC unit will be sent to the ICU were a staff physician will initiate the transfusion. Duration of transfusion will be standardized to 15 minutes in all patients, which will be supervised by a study investigator. The health care team, study participants and all study investigators remain blinded to treatment allocation status until final statistical analysis. Study investigators will be unblinded after enrollment of the last patient and completion of data collection.

6.3 Data and sample collection

- Age, sex and race of patients,
- size and weight of patients,
- reason for cardiac sugery ,
- co-existing morbidities,
- recent medication,
- laboratory values on the day of study,
- respiratory support and ventilator setting,
- APACHE II Score,
- duration of surgery,
- duration of cardiac bypass
- blood gas analysis immediately prior to and after transfusion, and 45 minutes following transfusion (T=0, 15, 60 min),
- vital signs immediately prior to, and every 5 minutes during transfusion, and 0, 15, 30 and 45 min after transfusion (T=0, 5, 10, 15, 30, 45, 60 min)
- hemodynamic data immediately prior to, and every 5 minutes during transfusion, and 0, 15, 30 and 45 min after transfusion (T=0, 5, 10, 15, 30, 45, 60 min)

- samples will be collected from arterial blood immediately prior transfusion (BL), immediately following transfusion (T15), 60 minutes after baseline (T60), and from each unit of PRBC immediately after transfusion.
- analysis of following laboratory parameters immediately prior to and after transfusion, and 45 minutes after transfusion: (T=0, 15, 60 min): erythrocyte count, hemoglobin, leukocytes, thrombocytes, serum glucose, creatine, BUN, GOT, GPT, AP, Na, K, bilirubin, free hemoglobin, syndecan-1 and MIF.

7 Statistics

7.1 Sample size calculation

Previous investigations have demonstrated a PAP increase of 1 ± 3 mmHg during transfusion of fresh PRBC, and of 4 ± 4 mmHg during transfusion of stored PRBC in lambs[9]. A sample size of 29 in each group has 80% power to detect a difference in means of 3 assuming that the common standard deviation is 4 using a two group t-test with a 0,05 two-sided significance level. Based on this and an expected drop-out rate of 10% we plan to include 64 patients in the study.

7.2 Hypothesis

We hypothesize that the increase in PAP and PVRI will be greater after transfusion of stored PRBCs than after transfusion of fresh PRBCs. In contrast, we expact no increase of PAP and PVRI after transfusion of fresh PRBCs.

7.3 Statistical analysis

The primary endpoint is the increase in PAP during the first 15 minutes after transfusion of PRBCs (ΔPAP15). In order to test whether he increase in PAP and PVRI will be greater after transfusion of stored PRBCs than after transfusion of fresh PRBCs t, a Welch's t-Test was conducted. Additionally, an ANCOVA using the baseline value, PAP0, as a covariate was computed and results were compared to those of the Welch's t-Test.

The secondary endpoints include:

- The increase in PAP during the first 60 minutes (ΔPAP60) and the increase in PVR, MAP, SVR, FHB and CO in the first 15 and 60 minutes (ΔPVR15, ΔPVR60, ΔMAP15, ΔMAP60, ΔSVR15, ΔSVR60, ΔFHB15, ΔFHB60, ΔCO15, ΔCO60). In order to test whether these parameters differ significantly between fresh and stored PRBCs, Welch's t-Tests were conducted.
- The correlation between ΔFHB15, ΔMIF15, FHB EK, MIF EK and ΔPAP15. The correlation was calculated using Pearson's formula and tested for significance using a t-Test.
- MIF EK, SDC EK, MIF0, SDC0, MIF15, SDC15, MIF60, SDC60, FHB0. In order to test whether these parameters differ significantly between fresh and stored

PRBCs, Welch's t-Tests were conducted. In order to test whether MIF15 differs significantly from MIF0 and SDC15 differs significantly from SDC0, dependent t-Tests with respect to the PRBC age were conducted.

- ΔFHB15, ΔFHB60, ΔMIF15, ΔMIF60, ΔSDC15, ΔSDC15. In order to test whether these parameters differ significantly from 0 (i.e. e.g. FHB15 differs significantly from FHB0), one sample t-Tests were conducted.

A value of $P<0.05$ will be considered statistically significant.

8 Data protection

All patients will be consecutively numbered and anonymized. Analyses will be performed anonymized according to the patient number. Only authorized study personnel will have access to data. Anonymized data will be stored and analyzed on computers with restricted access.

9 Ethical considerations and risk-benefit-evaluation

Transfusion of PRBCs will be part of patients' standard therapy. The included and randomized patients do not have any direct benefit from participating in this study. We do not expect that the randomized patients will be exposed to an increased health risk after receiving stored PRBCs instead of fresh PRBCs.

10 References

1. Bennett-Guerrero E, Veldman TH, Doctor A, Telen MJ, Ortel TL, Reid TS, Mulherin MA, Zhu H, Buck RD, Califf RM, McMahon TJ: Evolution of adverse changes in stored RBCs. Proc Natl Acad Sci U S A 2007; 104:17063-8

2. Gevi F, D'Alessandro A, Rinalducci S, Zolla L: Alterations of red blood cell metabolome during cold liquid storage of erythrocyte concentrates in CPD-SAGM. J Proteomics 2012

3. Baron DM, Yu B, Lei C, Bagchi A, Beloiartsev A, Stowell CP, Steinbicker AU, Malhotra R, Bloch KD, Zapol WM: Pulmonary hypertension in lambs transfused with stored blood is prevented by breathing nitric oxide. Anesthesiology 2012; 116:637-47

4. Baron DM, Beloiartsev A, Nakagawa A, Martyn T, Stowell CS, Malhotra R, Mayeur C, Bloch KD, Zapol WM: Adverse Effects of Hemorrhagic Shock Resuscitation with Stored Blood are Ameliorated by Inhaled Nitric Oxide in Lambs. Crit Care Med 2013 in press

5. Berra L, Coppadoro A, Yu B, Lei C, Spagnolli E, Steinbicker AU, Bloch KD, Lin T, Sammy FY, Warren HS, Fernandez BO, Feelisch M, Dzik WH, Stowell CP, Zapol WM: Transfusion of stored autologous blood does not alter reactive hyperemia index in healthy volunteers. Anesthesiology 2012; 117:56-63

6. Wang X, Tanus-Santos JE, Reiter CD, Dejam A, Shiva S, Smith RD, Hogg N, Gladwin MT: Biological activity of nitric oxide in the plasmatic compartment. Proc Natl Acad Sci U S A 2004; 101:11477-82
