# Supplementary material for: Transfusion of standard-issue packed red blood cells induces pulmonary vasoconstriction in critically ill patients after cardiac surgery—A randomized, double-blinded, clinical trial
Source: PLoS One. 2019 Mar 11;14(3):e0213000. doi: 10.1371/journal.pone.0213000 (PMC6411146; doi:10.1371/journal.pone.0213000)
Supplement: S2 Study Protocol — (PDF) [file pone.0213000.s006.pdf]

MEDIZINISCHE UNIVERSITÄT WIEN

Hämodynamische Auswirkungen der Transfusion gelagerter Erythrozytenkonzentrate bei Intensivpatienten

Dr. David Baron, Dr. Joanna Stefaniak und Ao.Univ.-Prof.Dr. Roman Ullrich

Universitätsklinik für Anästhesie, allgemeine Intensivmedizin und Schmerztherapie

Ass.Prof.Dr. Gerda Leitner

Universitätsklinik für Blutgruppenserologie und Transfusionsmedizin

Mag. Irene Steiner

Institut für Medizinische Statistik

Inhaltsverzeichnis

1 Hintergrund 2
2 Ziel 2
3 Patienten 3
4 Definition der Studiengruppen 3
5 Parameter 4
5.1 Hauptzielparameter 4
5.2 „Key secondary parameter“ 4
5.3 Nebenzielparameter 4
6 Methodik 5
6.1 Randomisierung und Verblindung 5
6.2 Datenerhebung 5
7 Statistik 7
7.1 Fallzahlplanung 7
7.2 Hypothesen 7
7.3 Statistische Methodik 8
8 Datenschutz 10
9 Nutzen-Risiko-Evaluierung 10
10 Referenzen 11

1 Hintergrund

Erythrozytenkonzentrate (EKs) dürfen vor ihrer Verabreichung für einen Zeitraum von maximal 42 Tagen gelagert werden. Während dieser Zeit finden in den Erythrozyten biochemische, pathophysiologische und funktionelle Veränderungen statt.[1,2]

Schafe haben im Vergleich zum Menschen eine ähnliche Anatomie und Physiologie der Lunge. Im Schafmodell konnte gezeigt werden, dass die Transfusion von 40 Tage lang gelagerten („alten") EKs eine pulmonale Hypertonie hervorruft,[3] während die Transfusion von 2 Tage lang gelagerten („frischen") EKs keine hämodynamischen Veränderungen bewirkte. Diese pulmonale Hypertonie während der Transfusion alter EKs wurde durch vorherige Induktion eines hämorrhagischen Schocks potenziert.[4]

Weiters zeigten Berra et al. in gesunden menschlichen Probanden, dass freies Hämoglobin nach Transfusion von 40 Tage alten EKs höher war als nach Transfusion von 3 Tage alten EKs.[5]

In dieser Studie soll untersucht werden, wie sich die Transfusion von unterschiedlich lange gelagerten EKs auf hämodynamische Parameter (pulmonaler und systemischer arterieller Druck, pulmonaler und systemischer Gefäßwiderstand, Herzzeitvolumen) von Intensivpatienten auswirkt. Die EKs werden nur dann transfundiert, wenn sie im Rahmen der klinischen Normalversorgung zur Therapie einer Anämie indiziert sind.

2 Ziel

Diese Studie soll zeigen, ob die im Schafmodell gemessene pulmonale Hypertonie und Vasokonstriktion während der Transfusion alter EKs auch im Menschen auftreten.

Sollte sich unsere Hypothese bestätigen, können diese Ergebnisse die gängigen Transfusionspraktiken verändern und dadurch die perioperative Betreuung kritisch kranker Patienten verbessern.

3 Patienten

Alle Patienten, die im Rahmen der Standardtherapie einen pulmonalarteriellen Katheter gelegt bekommen und auf einer Intensivstation der Universitätsklinik für Anästhesie, allgemeinen Intensivmedizin und Schmerztherapie behandelt werden, werden für den Einschluss in die Studie evaluiert. Patienten werden in die Studie eingeschlossen, wenn sie im Rahmen der Standardtherapie ein EK zur Behandlung der Anämie benötigen.

Ausschlusskriterien:

- Alter <18 Jahre
- Auf klinischer Erfahrung beruhende Vorhersage, dass der Patient die nächsten 24 h nicht überleben wird
- Akute Blutung mit Transfusionsbedarf von > 2 EKs/h
- Hoher Katecholaminbedarf (Noradrenalin > 0.2 µg/kg/min, jegliche Adrenalingabe)
- Therapie mit inhalativem Stickstoffmonoxid, inhalativem Prostazyklin oder Phosphodiesterase-5-Hemmern bis zu 24 h vor Einschluss die Studie
- Sepsis

4 Definition der Studiengruppen

Im Rahmend der Studie erfolgt eine prospektive Analyse der hämodynamischen Parameter während und nach der Transfusion von EKs bei kritisch kranken Intensivpatienten. Die während dieser Studie durchgeführten Maßnahmen sind Teil der normalen Patientenbetreuung und intensivmedizinischen Therapie. Der einzige Unterschied zu den gängigen Abläufen besteht darin, dass vor der Transfusion festgelegt wird, wie alt die EKs sind, die zur Transfusion herangezogen werden. Keines der transfundierten EKs wird die zugelassene Lagerungsdauer von 42 Tagen überschreiten.

„Frische“ EKs: Lagerungsdauer ≤ 14 Tage.

„Alte“ EKs: Standardtherapie („first in, first out“: das älteste in der Blutbank für den jeweiligen Patienten kompatible EK).

5 Parameter

5.1 Hauptzielparameter

- Vergleich der beiden Gruppen in Bezug auf die Differenz des pulmonalarteriellen Drucks zwischen Beginn (T=0 min) und Ende (T=15 min) der Transfusion.

5.2 „Key secondary parameter“

- Vergleich der beiden Gruppen in Bezug auf die Differenz des pulmonalarteriellen Widerstandes zwischen Beginn und Ende der Transfusion.
- Korrelation der Differenz des pulmonalarteriellen Druckes mit der Differenz der freien Hämoglobinkonzentration aller eingeschlossenen Patienten.

5.3 Nebenzielparameter

- Vergleich der beiden Gruppen in Bezug auf die Differenz des systemischen arteriellen Drucks und Gefäßwiderstands, sowie des Herzzeitvolumens zwischen Beginn und Ende der Transfusion.
- Vergleich der beiden Gruppen in Bezug auf die Änderungen der freien Hämoglobinkonzentration zwischen Beginn und Ende der Transfusion.
- Vergleich der beiden Gruppen in Bezug auf die Änderungen folgender Laborparameter: Erythrozyten, Hämatokrit, Hämoglobin, Leukozyten, Thrombozyten, Glucose, Kreatinin, BUN, GOT, GPT, AP, Na, K, Gesamtbilirubin.
- Nachverfolgung der oben genannten Parameter bis zu 45 min nach Beeindigung der Transfusion, um den zeitlichen Verlauf eventueller Veränderungen zu erheben.
- Sollten der Hauptzielparameter oder einer der „key secondary parameter“ positiv sein, wird zusätzlich ein sogenannter „Nitric oxide consumption assay“ durchgeführt (siehe „6.2 Datenerhebung“).[6]

6 Methodik

6.1 Randomisierung und Verblindung

Die Randomisierung und Verblindung erfolgt an der Universitätsklinik für Blutgruppenserologie und Transfusionsmedizin. Zwecks Randomisierung werden fortlaufend nummerierte, blickdichte Kuverts verwendet, welche die Zuteilung des jeweiligen Patienten zu einer der beiden Studiengruppen beinhalten. Die Randomisierung erfolgt dadurch, dass jenes Kuvert geöffnet wird, welches mit der dem Patienten zugewiesenen Nummer versehen ist.

Nach der Randomisierung des Patienten wird eine Konserve der zugewiesenen Lagerungsdauer ausgekreuzt und mit einer Klebeetikette versehen. Diese Klebeetikette ist mit der Nummer des Patienten versehen, und verdeckt zusätzlich das Ablaufdatum der EK. Dadurch wird gewährleistet, dass der behandelnde Arzt bezüglich der Lagerungsdauer der Konserve verblindet ist.

Sollte ein Patient, welcher in die Studie eingeschlossen und randomisiert wurde, kein EK benötigen, wird die Fehlrandomisierung vermerkt und ein Patient zusätzlich in die Studie eingeschlossen und randomisiert.

Die Entblindung erfolgt nach Einschluss und Datenerhebung des letzten Patienten in die Studie.

6.2 Datenerhebung

- Alter, Geschlecht und Rasse der Patienten,
- Größe und Gewicht der Patienten,
- Grund für Einschwemmen des pulmonalarteriellen Swan-Ganz Katheters,
- Grunderkrankung,
- Begleiterkrankungen,
- Laufende Medikation am Tag der Studie,
- Labor am Tag der Studie,
- Beatmungsform und -einstellungen,
- APACHE II Score,
- Operationsdauer (wenn Operation direkt vor Aufnahme auf Intensivstation),
- Anzahl und Alter der EKs, welche im Zeitraum von 2 Tagen vor der Studie verabreicht worden sind,

- Blutgasanalyse direkt vor und nach der Transfusion und 45 min nach der Transfusion (T=0, 15, 60 min),
- Vitalparameter direkt vor, alle 5 min während, und 0, 15, 30 und 45 min nach Ende der Transfusion (T=0, 5, 10, 15, 30, 45, 60 min),
- Hämodynamische Daten direkt vor, alle 5 min während, und 0, 15, 30 und 45 min nach Ende der Transfusion (T=0, 5, 10, 15, 30, 45, 60 min),
- Analyse der nachfolgenden Parameter direkt vor und nach der Transfusion und 45 min nach der Transfusion (T=0, 15, 60 min; jeweils 2+2 ml Blut): Erythrozyten, Hämatokrit, Hämoglobin, Leukozyten, Thrombozyten, Glucose, Kreatinin, BUN, GOT, GPT, AP, Na, K, Gesamtbilirubin.
- Sollte der Unterschied des Hauptparameters oder eines „secondary key parameter" statistisch signifikant sein, wird ein „Nitric oxide consumtion assay" (Stickstoffmonoxid-Verbrauch) durchgeführt: Blutplasma des Patienten wird direkt am Ende der Transfusion abgenommen (4 ml Blut), zentrifugiert und das gewonnene Plama bei -80°C gelagert. Die Plasmaproben werden dann in einem Chemilumineszenzdetektor mit bekannten Konzentrationen des Stickstoffmonoxiddonors Deta-NONOate inkubiert. Die daraus resultierenden Spannungsänderungen können in Stickstoffmonoxidverbrauch der einzelnen Plasmaproben umgerechnet werden.[6]

7 Statistik

7.1 Fallzahlplanung

Unsere Annahmen basieren auf experimentellen Versuchen in Schafen, welche eine ähnliche Anatomie und Physiologie der Lunge aufweisen wie der Mensch. In den Schafversuchen wurden umgerechnet zwei EKs innerhalb von 30 min transfundiert. Dabei stieg der pulmonalerterielle Druck während der Transfusion von „frischen" EKs um 1+/-1 mmHg und während jener von „alten" EKs 4+/-1 mmHg an.[3]

In der vorliegenden Studie wird nur ein EK über einen Zeitraum von 15 min verabreicht, weshalb wir von geringeren Streuungen ausgehen als in den Schafexperimenten. Wir erwarten folgende Änderungen während der Transfusion eines EK über 15 min:

„Frische" EKs: Anstieg des pulmonalarteriellen Drucks von Beginn (T=0 min) bis Ende (T=15 min) der Transfusion von 1+/-3 mmHg

„Alte" EKs: Anstieg des pulmonalarteriellen Ducks von Beginn (T=0 min) bis Ende (T=15 min) der Transfusion von 4+/-4 mmHg

Basierend auf einem zweiseitigen 2-Stichproben-t-Test werden 29 Patienten pro Gruppe benötigt um einen Unterschied von 3 mmHg zwischen den Gruppen bei einer Standardabweichung von 4 mmHg mit einer Power von 80% entdecken zu können (alpha = 5%).

Die Patienten werden aus der Studie ausgeschlossen, wenn während der Studiendauer eine akute Blutung mit Transfusionsbedarf von > 2 EKs/h auftritt, ein hoher Katecholaminbedarf (Noradrenalin > 0.2 µg/kg/min, jegliche Adrenalingabe) notwendig wird, oder die Patienten während der Studie versterben.

Bei einer erwarteten Drop-out-Quote von 10% wollen wir 32 Patienten pro Gruppe einschließen. Die Fallzahlplanung wurde mit NQuery 6.01 durchgeführt.

7.2 Hypothesen

Unsere Hypothese lautet, dass die Transfusion „alter" EKs eine Erhöhung des pulmonalarteriellen Drucks und Gefäßwiderstandes bei Intensivpatienten hervorruft. Weiters nehmen wir an, dass die Transfusion „frischer" EKs keine relevante

Erhöhung des pulmonalarteriellen Drucks und Gefäßwiderstandes in diesen Patienten hervorruft.

7.3 Statistische Methodik

Analyse des Hauptzielparameters

Um die Änderung des pulmonalarteriellen Drucks zwischen den beiden Gruppen zu vergleichen, wird ein lineares Modell berechnet, das die erwarteten ungleichen Varianzen in den beiden Gruppen berücksichtigt. Die abhängige Variable ist die Differenz des pulmonalarteriellen Drucks zwischen Beginn (T=0 min) und Ende (T=15 min) der Transfusion. Als unabhängige Variablen werden der Baseline-Wert, sowie die Gruppe (frische vs. alte EKs) in das Modell aufgenommen.

Analyse der „key secondary parameter“

Die Analyse der Differenz des pulmonalarteriellen Widerstands zwischen Beginn und Ende der Transfusion erfolgt analog der Analyse des primären Endpunktes.

Der Zusammenhang zwischen der Differenz des pulmonalarteriellen Drucks zwischen Beginn und Ende der Transfusion und der Differenz der freien Hämoglobinkonzentration zwischen Beginn und Ende der Transfusion wird mit einer Pearson Korrelation getrennt nach Gruppen analysiert.

Analyse der Nebenzielparameter

Die Analyse der sekundären Endpunkte hat explorativen Charakter. Die Analyse der Differenz des systemischen arteriellen Drucks und Gefäßwiderstands, des Herzzeitvolumens, sowie der Hämoglobinkonzentration zwischen Beginn und Ende der Transfusion erfolgt analog der Analyse des Hauptzielparameters.

Um den zeitlichen Verlauf des pulmonalarteriellen Drucks bis zu 45 Minuten nach der Transfusion zu analysieren, wird ein mixed model berechnet mit dem pulmonalarteriellen Druck 0, 15, 30 und 45 Minuten nach Ende der Transfusion als abhängige Variable. Als unabhängige Variablen werden der Baseline-Wert, Gruppe (frische vs. alte EKs), Zeit, sowie die Interaktion zwischen Zeit und Gruppe in das Modell aufgenommen. Die Patienten-ID wird als Zufallsfaktor im Modell berücksichtigt. Der Vergleich der einzelnen Zeitpunkte erfolgt durch Kontraste. Zusätzlich werden individuelle und mittlere Verlaufskurven pro Gruppe grafisch

dargestellt und deskriptive Statistiken (Minimum, Maximum, Quartile, Mittelwert, Standardabweichung) getrennt nach Gruppe und Zeitpunkt berechnet.

Die Analyse des zeitlichen Verlaufs des pulmonalarteriellen Widerstands, des systemischen arteriellen Drucks, des Gefäßwiderstands, des Herzzeitvolumens, sowie der Hämoglobinkonzentration erfolgen analog der Analyse des zeitlichen Verlaufs des pulmonalarteriellen Drucks.

Der Stickstoffmonoxidverbrauch der einzelnen Plasmaproben (Nitric oxid consumption assay) wird mit deskriptiven Statistiken (Minimum, Maximum, Quartile, Mittelwert, Standardabweichung) getrennt nach Gruppe analysiert.

Für alle statistischen Analysen wird das Signifikanzniveau auf 5% (2-seitig) gesetzt.

8 Datenschutz

Alle Patienten werden fortlaufend nummeriert und anonymisiert. Die Analysen erfolgen anonymisiert bezogen auf die Probandennummer. Nur autorisierte Personen haben Zugriff auf die Originaldaten Die anonymisierten Daten werden auf einem PC mit Zugriffsbeschränkung an der Universitätsklinik für Anästhesie, allgemeine Intensivmedizin und Schmerztherapie bzw. an der Universitätsklinik für Blutgruppenserologie und Transfusionsmedizin gespeichert und ausgewertet.

9 Nutzen-Risiko-Evaluierung

Die eingeschlossenen Patienten haben keinen direkten Nutzen von der Studie.
Das Risiko des Bekanntwerdens der sensiblen Patientendaten wird durch die Anonymisierung und Zugriffsbeschränkung minimiert. Es besteht kein zusätzliches Risiko für die Patienten, da alle Interventionen im Zuge der Standardtherapie erfolgen.

10 Referenzen

1. Bennett-Guerrero E, Veldman TH, Doctor A, Telen MJ, Ortel TL, Reid TS, Mulherin MA, Zhu H, Buck RD, Califf RM, McMahon TJ: Evolution of adverse changes in stored RBCs. Proc Natl Acad Sci U S A 2007; 104:17063-8

2. Gevi F, D'Alessandro A, Rinalducci S, Zolla L: Alterations of red blood cell metabolome during cold liquid storage of erythrocyte concentrates in CPD-SAGM. J Proteomics 2012

3. Baron DM, Yu B, Lei C, Bagchi A, Beloiartsev A, Stowell CP, Steinbicker AU, Malhotra R, Bloch KD, Zapol WM: Pulmonary hypertension in lambs transfused with stored blood is prevented by breathing nitric oxide. Anesthesiology 2012; 116:637-47

4. Baron DM, Beloiartsev A, Nakagawa A, Martyn T, Stowell CS, Malhotra R, Mayeur C, Bloch KD, Zapol WM: Adverse Effects of Hemorrhagic Shock Resuscitation with Stored Blood are Ameliorated by Inhaled Nitric Oxide in Lambs. Crit Care Med 2013 in press

5. Berra L, Coppadoro A, Yu B, Lei C, Spagnolli E, Steinbicker AU, Bloch KD, Lin T, Sammy FY, Warren HS, Fernandez BO, Feelisch M, Dzik WH, Stowell CP, Zapol WM: Transfusion of stored autologous blood does not alter reactive hyperemia index in healthy volunteers. Anesthesiology 2012; 117:56-63

6. Wang X, Tanus-Santos JE, Reiter CD, Dejam A, Shiva S, Smith RD, Hogg N, Gladwin MT: Biological activity of nitric oxide in the plasmatic compartment. Proc Natl Acad Sci U S A 2004; 101:11477-82
